# Therapeutic Potential of Photobiomodulation in Early Recovery After Experimental Spinal Cord Injury in Rats: Histological and Biomechanical Analysis

**DOI:** 10.1002/jbio.202500348

**Published:** 2025-12-15

**Authors:** Débora Campos Chaves Correia, Leonardo Borges de Lima, Mário Oliveira Lima, Luis Filipe Karatanasov Beloni, Raduan Hage, Emilia Angela Lo Schiavo Arisawa

**Affiliations:** ^1^ Biostimulation and Tissue Repair Laboratory Universidade do Vale do Paraíba—UNIVAP São José dos Campos Brazil; ^2^ Sensorimotor Rehabilitation Engineering Laboratory Universidade do Vale do Paraíba—Univap São José dos Campos Brazil; ^3^ Small Animal Veterinary Clinic Universidade do Vale do Paraíba—Univap São José dos Campos Brazil

**Keywords:** movement, nervous system, neuroprotection, photobiomodulation, spinal cord injury

## Abstract

Spinal cord injury (SCI) leads to severe functional deficits, underscoring the critical need for new therapies. This study evaluated the efficacy of photobiomodulation (PBM) as an early, noninvasive treatment for induced SCI, using specific parameters (808 nm, 72 J/cm^2^, 100 mW). A total of 15 rats were divided into Control (C), SCI, and PBM groups. Efficacy was determined by an integrated approach, correlating ground reaction force (GRF) with quantitative histological assessment. The PBM group showed a significant reduction of secondary damage (33.3 ± 5.5 vs. 53.9 ± 7.0 in the SCI group; *p* = 0.0002) and preserved neural structure. This tissue preservation aligns with the GRF analysis, which demonstrated that the PBM group recovered gait patterns similar to the Control group. In conclusion, PBM effectively mitigates necrosis aerea, maintaining tissue integrity, improving functional recovery, and reinforcing the PBM's therapeutic potential as a promising translatable strategy for outcomes after SCI. Further research should include immunostaining of cells and larger samples.

## Graphical abstract

Spinal cord injuries (SCI) cause severe motor and sensory deficits, with limited treatment options. This study demonstrates that early application of photobiomodulation (PBM) can protect spinal cord tissue and accelerate functional recovery in a preclinical model, which integrates biomechanical analysis with histomorphological characteristics of the tissue post‐SCI. The results showed that PBM preserves neural structures, reduces tissue damage, and improves gait performance, highlighting PBM as a safe, noninvasive therapy with potential to enhance recovery after SCI in future translational research.

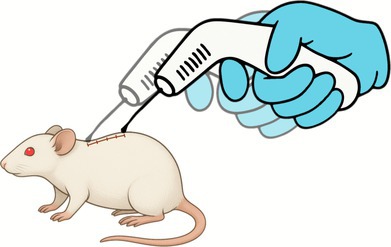

## 1 Introduction

Spinal cord injury (SCI) is characterized by the interruption of neural pathways in the spinal cord, often resulting in considerable sensorimotor deficits, compromised autonomic regulation, and distinct levels of temporary or permanent disability [1]. The global incidence of SCI has increased over the last few decades, primarily associated with traffic accidents and falls [2, 3, 4]. The global incidence ranges from 10.4 to 83 cases per million people per year, depending on the country and data collection methods used. Approximately 12 000 new cases are reported annually in the United States, and the medical expenses, rehabilitation, and loss of productivity can exceed $1 million per person. In Brazil, although there is a lack of accurate national data on the incidence of SCI, it is estimated that 12–18 million people live with some form of physical disability. According to the Brazilian Institute of Geography and Statistics (IBGE), approximately 1.3% of the population has some form of severe motor disability [5, 6, 7, 8].

The severity and anatomical level of SCI significantly influence neurological recovery. Thoracic and penetrating spinal cord injuries are more likely to result in complete injury, with less expectation of functional recovery [9]. Motor and sensory deficits arise due to the interruption of descending and ascending neural pathways formed by motor and sensory axons [10]. Experimental studies using animal models are conducted to understand the histopathological processes and motor impairment of SCI, validated by three types of SCI models: transection, contusion, and compression [11]. Among these, contusion SCI is a particularly valuable model for studying axonal growth dynamics, glial scar formation, and inflammatory response [3, 10, 12, 13, 14].

SCI triggers immediate and widespread death of nerve cells at the site of injury due to mechanical forces that compromise tissue structure, especially the gray matter. Axonal rupture disrupts communication between the brain and peripheral nerves, leading to paralysis and sensory loss. Hypoxia and inflammation exacerbate tissue damage over time, further limiting tissue repair [15, 16, 17]. Additional secondary damage, including oxidative stress and apoptosis, restricts the effectiveness of regenerative processes [1, 18, 19].

The impact of SCI extends beyond neurological deficits, affecting the quality of life and independence, particularly in daily activities and work disability. The social and economic burden of SCI is exacerbated by the severity of the injury and the resulting work disability. In addition, the costs associated with the SCI treatment and long‐term support to maintain a minimum quality of life for patients are substantial [20].

SCI treatment remains a major challenge in healthcare, despite extensive research efforts. Current therapeutic strategies, which involve surgical procedures, systemic pharmacological treatments, and extended physical therapy regimens, demonstrate limited clinical success rates. This highlights a critical imperative for systematic development of innovative therapeutic approaches to bridge the identified knowledge deficits, especially given the unresolved scientific and translational gaps [21, 22]. Several treatment protocols have been proposed using experimental models to improve and/or restore locomotor functions [14]. However, due to the multifaceted nature of SCI and the associated cellular and molecular alterations, efficient treatment requires the combination of complex therapeutic interventions to promote neuroregeneration [23]. Among the emerging therapeutic strategies, photobiomodulation (PBM) has gained attention for its potential to enhance neural recovery [24].

PBM is a noninvasive, nonthermal process that activates endogenous chromophores at different biological levels, inducing photophysical and photochemical events in injured tissues. This process produces therapeutic effects, including pain relief, inflammation reduction, immunomodulation, and the promotion of wound healing and tissue regeneration [25, 26, 27, 28, 29, 30, 31, 32].

Clinically, PBM has been applied with several treatment protocols for conditions such as skeletal muscle injuries [33, 34, 35], rheumatoid arthritis [36], and pain management [37, 38]. In addition, PBM has been investigated for neurological and neurodegenerative disorders, including traumatic brain injury [29, 39], multiple sclerosis, Alzheimer's, and Parkinson's disease [40]. In SCI models, researchers have explored transcutaneous laser therapy with various irradiation parameters and treatment durations to evaluate its effectiveness [41, 42]. Phototherapy, in the 600–900 nm range, has demonstrated the ability to stimulate cellular metabolic processes, a phenomenon that has been applied in the medical field [42, 43]. Among the therapeutic approaches investigated, PBM therapy stands out as a noninvasive technique that provides energy to viable cells, triggering tissue regeneration processes. Increasing evidence suggests that PBM has beneficial effects in the context of regenerative medicine, promoting the recovery of damaged tissues in different experimental models [44, 45, 46].

Histological analysis of injured tissues is widely used to assess the effects of therapeutic interventions in experimental models. The cavitation area stands out among histological parameters as a critical metric in central nervous system (CNS) injury studies. It directly quantifies the extent of tissue degeneration and permanent loss following SCI, thereby enabling accurate assessment of structural preservation and serving as the primary endpoint for comparative evaluation of treatment efficacy [47]. However, for a more comprehensive understanding of functional recovery, it is essential to associate these findings with biomechanical parameters. In this context, the analysis of ground reaction force (GRF) during gait emerges as a highly relevant complementary tool, as it allows for the objective assessment of the effectiveness of therapeutic protocols [48].

GRF is generated in response to the force applied by the limb against the ground and is one of the main parameters used in the biomechanical analysis of locomotion. This variable provides detailed information on weight distribution, balance, gait patterns, and functional changes resulting from injuries, surgical interventions, or rehabilitation treatments [48]. GRF analysis is performed to measure the force exerted by the ground at the moment of contact of the limb with the surface during locomotion, allowing the assessment of the functional locomotor recovery and complementing histological analysis, contributing to the validation of the results obtained.

The primary goal of this study was to integrate data derived from functional biomechanical assessments and microstructural histological evaluations to determine the therapeutic potential of PBM following contusion‐induced SCI. This approach provides a holistic understanding the PBM's effect on both structural preservation and functional recovery.

## 2 Experimental Procedures

Fifteen rats (*Rattus norvegicus albinus*, Wistar, male, 90 days old, weighing 330 ± 20 g) were housed in polypropylene plastic cages in a controlled temperature environment (20°C ± 2°C, 12/12 h light/dark cycles), with free access to food and water (Ethical Commission in Animal Use approval: A8‐CEA‐2022).

The animals were manually randomly allocated into three groups (*n* = 5): Control (C)—surgical procedures and simulation of SCI; Injury (SCI)—spinal cord injury without treatment; and PBM—SCI treated with a PBM protocol.

### 2.1 Surgical Procedures

Initially, the animals received the association of 50 mg/kg of ketamine hydrochloride (0.01 mL/kg, Agener, SP, Brazil), 5 mg/kg of xylazine hydrochloride (0.005 mL/kg, Dopaser, SP, Brazil), 5 mg/kg tramadol hydrochloride (5 mL/kg, Medley, SP, Brazil) and acepromazine (2 mL/kg, Vetnil, SP, Brazil). Sedation was maintained with continuous inhalation of isoflurane (1.5%) diluted in 100% O_2_ on an Isoflurane vaporizer (I21025, Ugo Basile S.R.L., Italy).

The surgical procedure was initiated after trichotomy and antisepsis with gauze soaked in liquid povidone of the dorsal region. An incision was made 7 cm from the base of the animal's ear, corresponding to the T9–T10 region of the rat's spinal cord.

**FIGURE 1:**
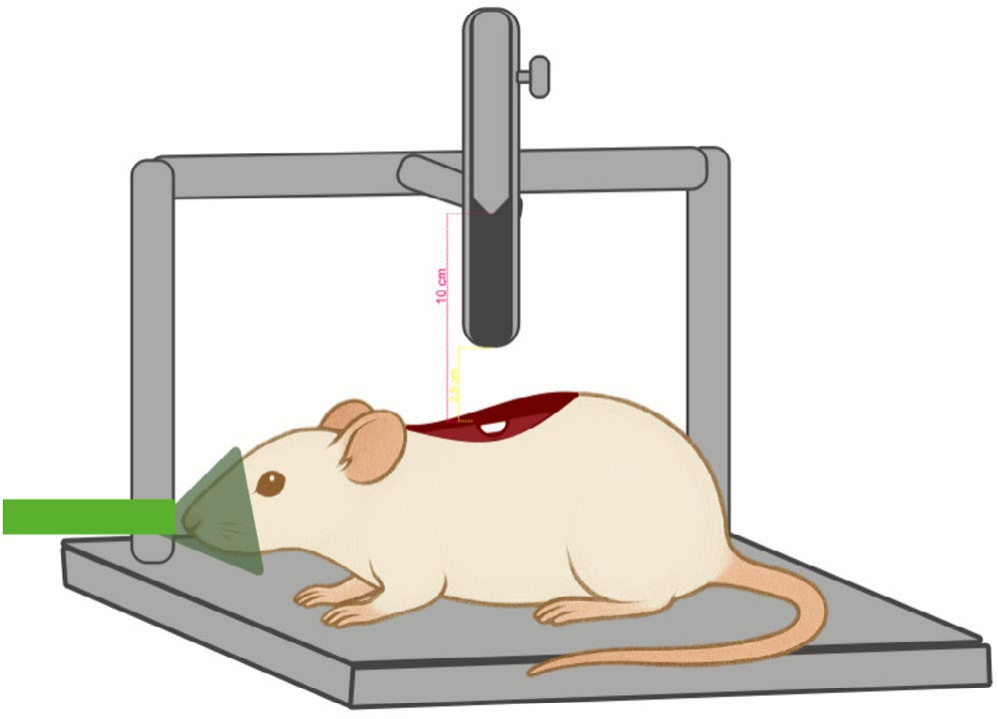
Preparatory steps for SCI induction. The procedure involves maintaining the rat under isoflurane inhalation anesthesia while the spinal cord is surgically exposed to facilitate the subsequent injury protocol.

The spinal cord was exposed using a laminectomy protocol, with a longitudinal skin incision (2.5 cm) in the dorsal midline, with a No. 3 scalpel and No. 15 blade. An incision was made in the dorsal fascia, with two longitudinal cuts parallel to the spinous processes of the vertebrae. The paravertebral muscles were displaced to expose the articular processes of the vertebrae. Using a punch and a gouge, the T9 and T10 spinous processes were carefully removed, exposing the spinal cord. SCI was induced in the I and PBM groups by direct trauma, with a free fall of a weight (10 g) attached to a mini guillotine, positioned at a height of 25 mm over the exposed spinal cord (Figure 1). The weight was removed 20 s after contact with the spinal cord [29, 49].

The Control group underwent only exposure of the spinous processes. After the surgical procedures, antibiotics (Pentakel, 0.01 mL/kg, intramuscularly), analgesics (tramadol hydrochloride, 5 mg/kg, intramuscularly), and dipyrone (20 mg/kg, orally) were administered at 12‐h intervals for 5 days.

### 2.2 PBM

**FIGURE 2:**
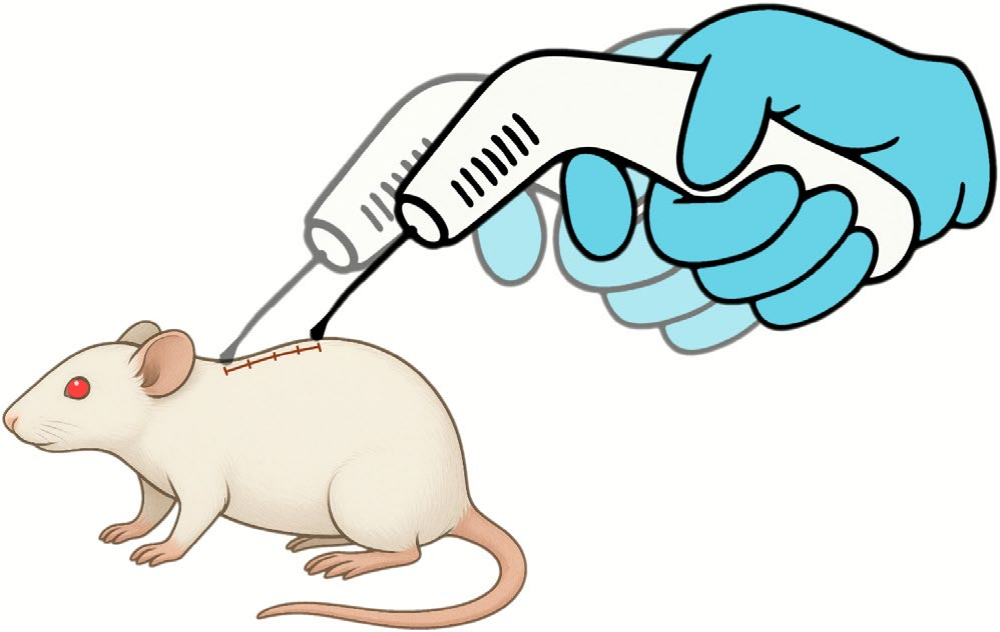
Diagram illustrating the precise anatomical points over the spinal cord injury site where photobiomodulation was delivered.

The PBM group underwent PBM therapy after SCI and on alternate days, transcutaneously and perpendicularly to the incision site, at two specific points (Figure 2). PBM was applied using Therapy EC equipment (DMC Equipamentos, SP, Brazil) using infrared irradiation (IR) following the protocol described in Table 1.

**TABLE 1:** PBM protocol applied in the present study.

Parameters	Laser (DMC, modelo therapy EC)
Wavelength (*λ*)	808 nm
Energy (*E*)	2 J
Radiant exposure (RE)	72 J/cm^2^
Radiant power (P)	100 mW (0.1 W)
Power density (PD)	3.6 W/cm^2^
Spot area (A)	0.028 cm^2^
Emission mode	Continuous
Irradiation form	Contact
Time (*T*)	20 s (per point)
Total energy	4 J
Frequency (days)	Alternate
Number of points	2

PBM was administered using an 808 nm (AlGaAs) diode laser (DMC, Therapy EC model) operating in continuous wave (CW) mode. The device delivered an output radiant power of 100 mW (0.1 W) across a spot size of 0.028 cm^2^, resulting in an irradiance of 3.6 W/cm^2^. Each irradiation point received a total energy of 2 J (a radiant exposure of 72 J/cm^2^) over an exposure time of 20 s using a contact mode application. Two points were treated per season, yielding a total energy of 4 J per session. The protocol was applied on alternate days across four seasons, achieving a cumulative dose of 16 J. The laser device was factory‐calibrated before the experiments, and no further adjustments were made during this study.

### 2.3 Biomechanical Analysis

**FIGURE 3:**
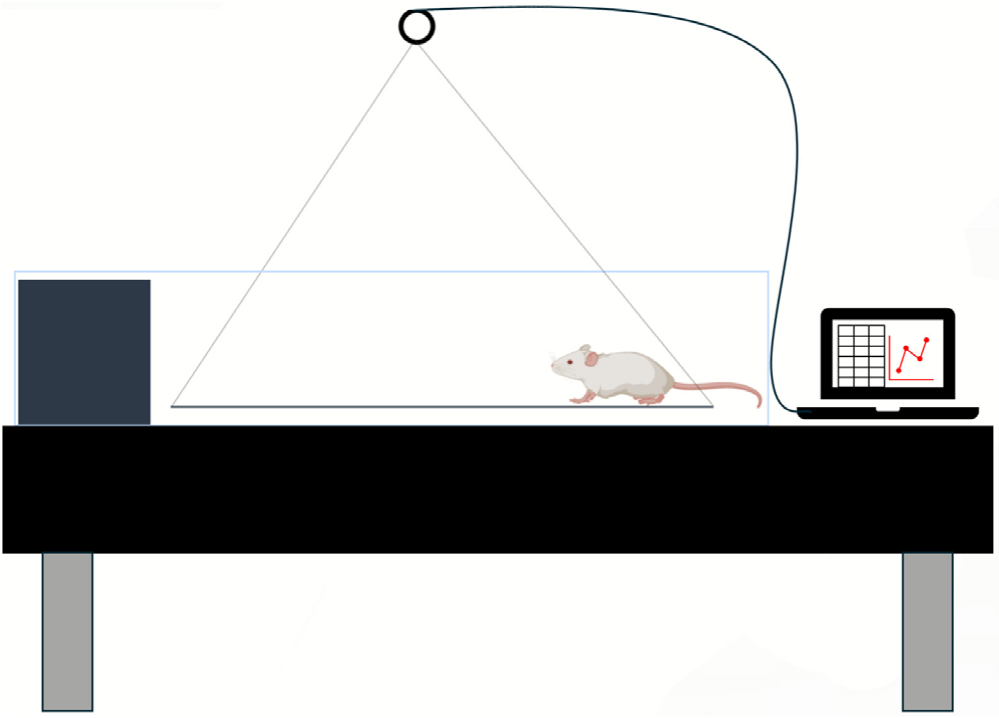
Schematic representation of the experimental setup for GRF.

Biomechanical assessment was performed through gait analysis, focusing on displacement time and plantar force distribution (PFD). GRF analysis was performed in a clear glass positioned on a NewPort SmartTable optical table. Voluntary sensorimotor force was measured using a suspension bridge device custom‐designed for this study. This setup incorporated a translucent acrylic plate (70 cm long), which was suspended by nylon cables connected, and connected to a high‐precision load cell (HBK, GLHX711, class C3, 0.05g resolution). The load cell, coupled to a 24‐bit HX711 amplifier/analog‐to‐digital converter and rigidly fixed to the table's suspended structure to isolate measurements from ambient vibrations (Figure 3). Force data were collected via a USB interface and subsequently processed in MATLAB to calculate PFD along the trajectory.

All animals were euthanized 7 days post‐injury using an anesthetic overdose. Consisted of ketamine hydrochloride (150 mg/kg, Agener, SP, Brazil) associated with xylazine hydrochloride (50 mg/kg, Dopaser, SP, Brazil) and acepromazine (20 mL/kg, Vetnil). Subsequently, the SCI region was surgically excised. The resulting tissue fragments were fixed in 10% buffered formalin for 24 h. The samples were washed and decalcified with ethylenediaminetetraacetic acid (EDTA, NEON) to remove calcium with minimal tissue alteration. The solution was changed every 2 days, corresponding to 10 changes [50].

**FIGURE 4:**
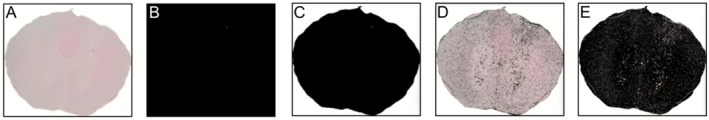
Illustration of the comprehensive area measurement technique applied to histological sections. The sequence demonstrates the steps for quantification (A) original tissue section; (B) measurement of the total image area; (C) measurement of the spinal cord cross‐sectional area; (D) identification and subsequent quantification of the cavitation areas (lesion sites), highlighted in black; and (E) identification and quantification of the preserved tissue areas, also initially highlighted in black, allowing for relative area calculations.

After tissue processing (dehydration, diafanization, and impregnation), the samples were embedded in paraffin and sectioned in histological sections (6 μm) using a semi‐automated rotary microtome (Leica HistoCore MULTICUT‐RM2245) with intervals of 10 per slide, stained with hematoxylin and eosin (H&E, Merck). The slides were analyzed under optical microscopy (Nikon Eclipse e200), and digital images were taken using a Leica DM 2500 microscope coupled to a Leica DFC 425 camera and Leica Application Suite Program v3.7. Quantification of tissue degeneration was obtained by measuring the cavitation area (Figure 4) using ImageJ software (National Institutes of Health, Bethesda, MD, USA) [47]. Data are expressed as mean ± SD with 95% CI. Statistical analyses involved a one‐way ANOVA for intra‐ and inter‐group comparisons, followed by a Tukey post hoc test to correct for multiple comparisons, with significance set *p* < 0.05. The sample size (*n* = 5 per group) was selected based on the 3R principles (reduction, refinement, and replacement) and Brazilian Resolution No. 55/2022, and based on the guidelines of Damy [51], ensuring robust results with ethical minimal animal commitment.

Analyses were conducted in Google Colab using the Pandas, NumPy, SciPy, and StatsModels libraries. Data visualization was performed using Matplotlib and Seaborn to generate box plots. These plots were specifically used to illustrate data distribution and facilitate the visual identification of statistical differences across the experimental groups.

## 3 Results

The GRF and histological image analyses presented marked differences in SCI healing between the groups, including functional recovery patterns and the presence of cavitation areas.

GRF analysis revealed notable differences, highlighting the beneficial effects of PBM therapy on gait restoration after SCI. This analysis allowed a dynamic assessment of injury and therapeutic results on gait throughout the observation period.

### 3.1 Qualitative Histological Analysis

#### 3.1.1 Control Group (C)

Histological analysis of H&E‐stained spinal cord tissue revealed the characteristic organization of the CNS. The white matter presented a network of nerve fibers and glial cells, while the gray matter contained numerous neuronal bodies, surrounded by glial cells, dendrites, and myelin‐covered axons.

#### 3.1.2 Injury Group (I)

SCI resulted in substantial histopathological changes in the specimens of this group. H&E‐stained sections revealed extensive areas of tissue cavitation, indicating pronounced tissue loss. This was accompanied by a marked reduction in the density of nerve fibers and neuronal and glial cells within the preserved parenchyma. Conversely, an increased cellularity (evidenced by numerous cell nuclei) was observed surrounding the lesion epicenter. This border region, largely composed of reactive glial cells, encircling the cavitation area, suggests a robust reactive gliosis in response to the preceding cellular necrosis.

#### 3.1.3 PBM Group (PBM)

**FIGURE 5:**
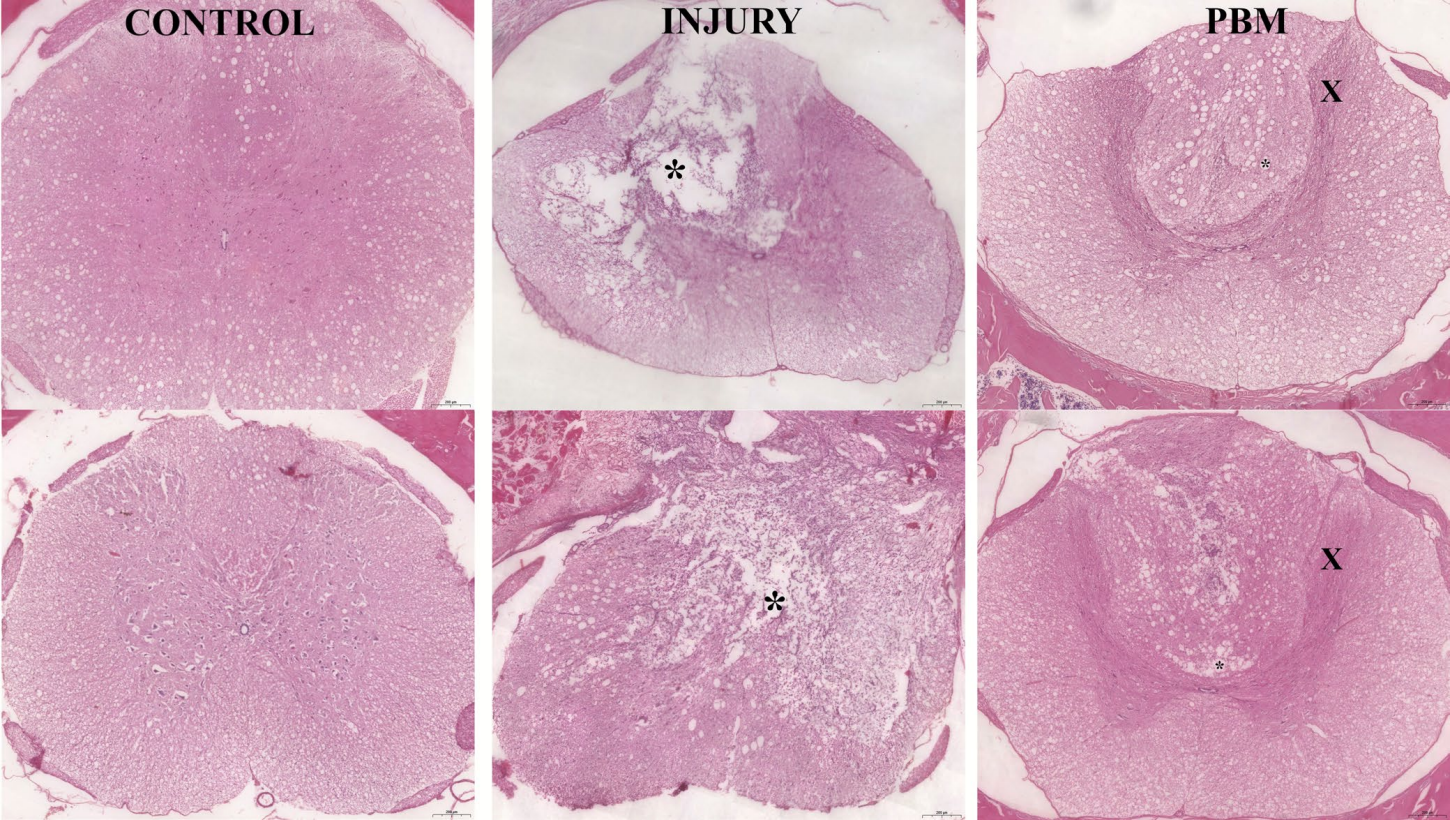
H&E‐stained histological photomicrographs (×20 magnification) comparing spinal cord morphology across the Control (C), Injury (I), and photobiomodulation (PBM) groups. Cavitation areas are indicated by asterisks (*), while “x” marks preserved nerve fibers. Scale bar = 200 μm.

Histological sections of the PBM group stained with H&E (Figure 5) revealed tissue preservation compared to the Injury group. Microcavities with defined contours, surrounded by cell nuclei, and an apparent attempt at reorganization of the nerve fibers in the injured area were observed. The areas of cavitation present in the white matter were reduced in size compared to those detected in the L group. In addition, numerous neuronal nuclei and glial cells were present in the gray matter.

### 3.2 Quantitative Histological Analysis

**FIGURE 6:**
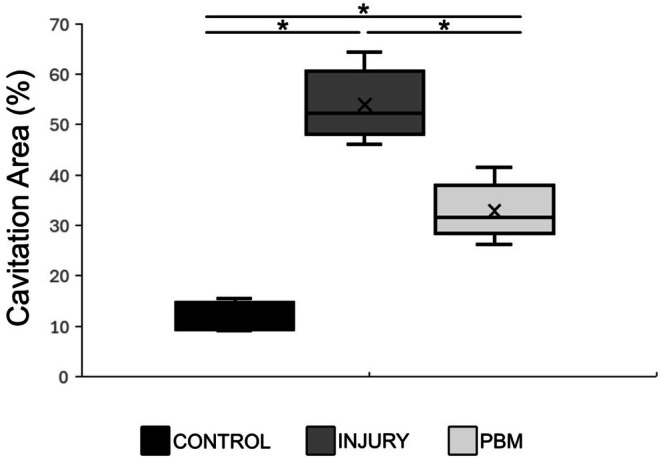
Quantitative analysis of cavitation area (%) across the Control (C), Injury (I), and photobiomodulation (PBM) groups. Data are presented using a boxplot (or specifically: mean ± SD or median with IQR). A significant difference between groups was assessed using one‐way ANOVA followed by Tukey's post hoc test. All pairwise group comparisons (C vs. I, C vs. PBM, and I vs. PBM) showed statistical significance (*p* < 0.05).

Quantitative histological analysis of the cavitation area, summarized in the boxplot (Figure 6), confirmed significant differences among the experimental groups, corroborating the qualitative observations. The Injury (I) group exhibited the highest median cavitation area, whereas the Control (C) group displayed the lowest. The post hoc Tukey test revealed highly significant differences between all comparisons: C versus I (11.97 ± 2.76 × 53.93 ± 7.08; *p < 0.01), I versus PBM (53.93 ± 7.08 × 33.35 ± 5.55; *p = 0.0002), and C versus PBM (11.97 ± 2.76 × 33.35 ± 5.55; *p = 0.0001).

Qualitative and quantitative analyses of the cavitation areas demonstrated a highly significant statistical difference among the groups. These findings permitted a clear classification based on the magnitude of permanent tissue loss: Injury (I) > PBM > Control (C). This descending order directly correlates with preserved tissue integrity in the SCI region, where the minimal cavitation areas in the PBM group signify a greater presence of viable neurons, glial cells, and intact nerve fibers.

### 3.3 GRF Analysis

**FIGURE 7:**
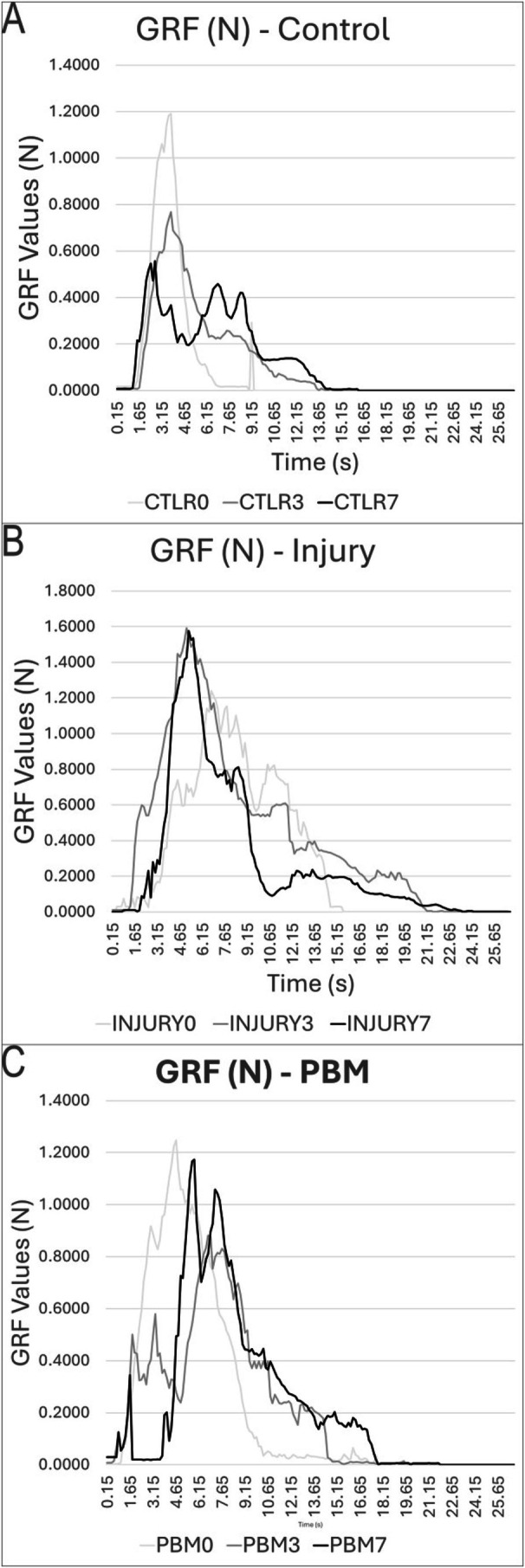
Ground reaction force, with control (A), injury (B), and PBM (C) graphs, showing the GRF by platform passage time, and in each of the graphs, the days of analysis are represented by different colors. Day 0, in light gray line, Day 3, dark gray line, and Day 7 in black line.

Graphs A–C (Figure 7) represent the GRF analysis during the stance phase of locomotion, illustrating the GRF over time, measured in Newtons (N). The vertical axis (*y*) represents the GRF magnitude, and the horizontal axis (*x*) represents the duration of ground contact (s). Each curve displays the mean GRF profile for the three experimental groups (C, I, and PBM) across different assessment time points: Days 0, 3, and 7. These data facilitate the analysis of load distribution and biomechanical behavior changes following the intervention.

The results obtained after collecting GRF data are shown in Table 2.

**TABLE 2:** GRF data per group at each experimental period.

DATA	CTLR	INJURY	PBM
GRF data on Day 0			
Mean	0.1016	0.2283	0.3211
Max	1.1903	1.2478	1.2401
Min	0.0166	0.0007	0.0287
Peak time	3.75	4.65	6.75
Travel time	9.15	18.3	14.55
GRF data on Day 3			
Mean	0.1178	0.4258	0.213
Max	0.7679	1.5911	0.8813
Min	0.0074	0.008	0.006
Peak time	3.75	5.1	6.75
Travel time	14.85	21.15	15.15
GRF data on Day 7			
Mean	0.121	0.2884	0.2307
Max	0.5572	1.576	1.1736
Min	0.0033	0.005	0.001
Peak time	2.7	5.25	5.85
Travel time	16.55	24.45	21.75

*Note:* Values expressed as mean. GRF values expressed in Newtons (N) and time expressed in seconds (s).

Abbreviations: CTLR, control group; INJURY, injury group; Max, maximum GRF value; Mean, mean GRF value; Min, minimum GRF value; PBM, photobiomodulation group; Peak time, time spent to reach maximum GRF value; Travel time, time spent to complete the path.

Analysis of the mean GRF (Table 2) revealed significant statistical differences for all inter‐group comparisons (C vs. I, C vs. PBM, and I vs. PBM). The *p* value for the overall comparisons was 0.0011, 0.024, and 0.0041, respectively. This significance was maintained throughout the time course: as Day 3 (C vs. I, *p* = 0.0030; C vs. PBM, *p* = 0.047; I vs. PBM, *p* = 0.0019) and at Day 7 (C vs. I, *p* = 0.0038; C vs. PBM, *p* = 0.0041; I vs. PBM, *p* = 0.0066). Functionally, the PBM group showed improved motor patterns: although the GRF peak time was delayed on Day 3, it significantly accelerated by Day 7 relative to the post‐SCI baseline. Consistent with functional improvement, PBM‐treated animals also demonstrated a reduced total time spent traveling compared to the Injury group.

## 4 Discussion

The PBM protocol utilized in this study provided compelling evidence of efficacy in mitigating the progression of secondary SCI. This effect was clearly evidenced by a marked reduction in the necrotic tissue area, indicating a significant neuroprotective benefit in sparing critical spinal cord tissue and limiting post‐contusion pathology. This structural preservation, particularly concerning neural and glial components, reinforces PBM's potential as a robust neuroprotective strategy in acute injury.

The persistent search for effective SCI treatments is driven by the inherent difficulty in treating central nervous tissue—a major challenge for patient recovery. The scarcity of established protocols and limited therapeutic advances in this area [44, 52] motivated our approach, which integrates movement analysis with detailed histomorphological characteristics of the central nervous tissue post‐SCI.

This preclinical study performed both qualitative and quantitative evaluations, correlating microscopic tissue characteristics with functional locomotor parameters assessed via movement analysis. The results collectively indicate significant positive effects with the PBM protocol compared to the untreated group. Histopathological analysis, the gold standard for evaluating tissue repair, provided crucial insights into the early phase of tissue repair, particularly through H&E staining.

The Control group (sham surgery) exhibited normal, healthy nervous tissue features. In stark contrast, the untreated Injury group (Group I) revealed large areas of tissue cavitation surrounded by a pronounced glial reaction. This demonstrated that SCI induces an intense inflammatory response, leading to rapid cellular degeneration and necrosis. These cavitation regions, typically encircled by reactive astrocytes and activated microglia [53, 54], result from the enzymatic degradation of the spinal cord extracellular matrix, including demyelination and axonal loss. The eventual formation of these areas causes permanent loss of sensory and motor functions [42].

The inflammatory response following SCI is characterized by a significant increase in resident microglia, which are responsible for phagocytizing necrotic cells, and the recruitment of peripheral immune cells, such as macrophages, when the blood–spinal cord barrier is compromised [1, 18, 55, 56, 57]. During the acute phase of SCI, reactive gliosis—the proliferation and activation of glial cells—significantly contributes to lesion progression. Glial activation drives secondary damage by releasing pro‐inflammatory mediators and, critically, by forming the glial scar, which physically isolates the damaged tissue and erects a major barrier to axonal regeneration. However, in later stages, these same cells assist in debris clearance and secrete trophic and anti‐inflammatory factors that promote tissue repair and structural reorganization [58]. The smaller size and reduced number of cavitation areas in the PBM‐treated group, when compared to Group I, suggest that the viable cells absorbed the phototherapy energy, as proposed by the mitochondrial mechanism of action [32, 42, 44, 59, 60], thereby maintaining cellular integrity and limiting the expansion of tissue loss.

The SCI model successfully induced loss of motor and sensory function, enabling a comparative analysis between the affected and preserved sides [33, 48]. The GRF analysis provides a clearer understanding of the functional progression. As expected, Control animals maintained stable GRF values, supporting the internal validity of the model. The untreated group showed a progressive functional deterioration, characterized by a decline in GRF and shorter stance duration over the 7‐day period.

Conversely, the PBM‐treated group exhibited a distinct pattern of functional recovery. While initially compromised, the GRF curves demonstrated progressive improvements on Days 3 and 7, with increases in both the amplitude and duration of force application. By Day 7, the GRF profile of the PBM group closely approached that of the Control group, indicating that the PBM‐treated group effectively contributed to the accelerated restoration of locomotor function. These functional improvements reinforce the histological findings that PBM is effective in improving biomechanical parameters by preserving underlying tissue. The therapeutic success of PBM is highly dependent on a precise dose–response relationship, requiring meticulous calibration of parameters [46, 48, 61, 62, 63, 64, 65, 66].

Our histological and GRF results converge to strongly reinforce the effectiveness of PBM in modulating the post‐SCI tissue response. This integrated evidence suggests that PBM application, under the described experimental conditions, limited the inflammatory cascade, thereby reducing the expansion of the cavitation areas and decreasing glial scar formation.

Given its characteristics as a relatively cost‐effective the long‐term, noninvasive, and side‐effect‐free therapy, the timely application of PBM following SCI has demonstrated promising therapeutic results. These compelling findings substantiate the multidimensional therapeutic effects of PBM, both structural and functional, and strongly encourage subsequent rigorous research aimed at validating this approach as a viable and promising strategy for SCI treatment.

We recognize that the simple size of five animas per group may limit the statistical power necessary to detect subtle therapeutic differences, particularly in secondary outcomes. While this approach adhered to 3R principles for animal welfare, the low N may increase bias. Future confirmatory studies should integrate a larger cohort to increase the robustness of the statistical findings. The absence of a true sham PBM group, receiving the same handling and laser apparatus exposure but with the laser beam inactive, could definitively exclude the possibility of nonspecific effects related purely to the handling, stress, or sensory input of the intervention procedure itself. Therefore, while our current data strongly support a photobiological mechanism, this study design limitation mandates the inclusion of a dedicated sham PBM group in subsequent, fully powered investigations to confirm the therapeutic effects. Future research will focus on detailed immunostaining of microglia and astrocytes to evaluate their roles in tissue necrosis and glial scar formation. The study will also extend the post‐SCI evaluation period to thoroughly assess long‐term functional and histological outcomes.

In summary, this study provides compelling evidence that the therapeutic efficacy of PBM is directly linked to the use of specific irradiation parameters. The protocol, utilizing a near‐infrared wavelength of 808 nm, an output power of 100 mW, and a total fluence of 72 J/cm^2^, successfully modulated the post‐injury inflammatory cascade, resulting in significant histological tissue preservation and functional locomotor improvements. These benefits were achieved without any observable adverse effects, reaffirming the safety and noninvasive nature of PBM at these low‐level energy settings. The absence of negative outcomes strengthens the rationale for advancing this specific protocol to further clinical investigation as a neuroprotective strategy for acute SCI.

## 5 Conclusion

This integrated study demonstrates the therapeutic efficacy of PBM (808 nm, 72 J/cm^2^, 100 mW) as a robust intervention for spinal cord injuries. Our findings establish that early PBM is a critical modulator of the post‐injury microenvironment, exerting significant neuroprotection by curbing secondary tissue damage and limiting glial scar formation in the acute phase. Crucially, the functional outcome, as rigorously quantified by GRF analysis, revealed a rapid and accelerated locomotor recovery, with treated GRF patterns nearing control levels just 7 days post‐injury, an unprecedented acceleration in functional return. Concurrently, histological data confirmed this effect, showing marked preservation of tissue integrity and reduced cavitation areas at the injury's epicenter. Collectively, these data substantiate PBM's potential to fundamentally shift the paradigm for SCI management, providing a noninvasive, high‐impact strategy for mitigating functional damage and rapidly accelerating functional recovery after SCI.

## Author Contributions

**Débora Campos Chaves Correia:** methodology, investigation, data curation, visualization, writing – original draft, writing – review and editing. **Leonardo Borges de Lima:** methodology, investigation, data curation, visualization, writing – original draft, writing – review and editing. **Mário Oliveira Lima:** methodology, investigation, validation, formal analysis, funding acquisition, data curation, visualization, writing – review and editing. **Luis Filipe Karatanasov Beloni:** methodology, investigation, writing – review and editing. **Raduan Hage:** methodology, investigation, validation, formal analysis, funding acquisition, writing – review and editing. **Emilia Angela Lo Schiavo Arisawa:** conceptualization, supervision, validation, formal analysis, funding acquisition, data curation, visualization, writing – original draft, writing – review and editing. All authors have read and agreed to the published version of the manuscript.

## Funding

The authors have nothing to report.

## Ethics Statement

The study was submitted to and approved by the Committee of Ethics in Animal Use (CEUA), registered under protocol numbers A8‐CEA‐2022. All institutional ethical guidelines and standards applicable to the care and use of animals were followed. This is not a clinical trial.

## Conflicts of Interest

The authors declare no conflicts of interest.

## Data Availability

The data that support the findings of this study are available on request from the corresponding author. The data are not publicly available due to privacy or ethical restrictions.

## References

[1] C. S. Ahuja , J. R. Wilson , S. Nori , et al., “Traumatic Spinal Cord Injury,” Nature Reviews Disease Primers 3 (2017): 17018. 10.1038/nrdp.2017.18 28447605

[2] E. M. Hagen , T. Rekand , N. I. Gilhus , and M. Grønning , “Traumatic Spinal Cord Injuries—Incidence, Mechanisms and Course,” Tidsskrift for den Norske Lægeforening 132, no. 7 (2012): 831–837. 10.4045/tidsskr.10.0859 22511097

[3] A. R. Brown and M. Martinez , “Thoracic Spinal Cord Hemisection Surgery and Open‐Field Locomotor Assessment in the Rat,” Journal of Visualized Experiments 148 (2019): e59738. 10.3791/59738 31305520

[4] W. Ding , S. Hu , P. Wang , et al., “Spinal Cord Injury: The Global Incidence, Prevalence, and Disability From the Global Burden of Disease Study 2019,” Spine 47, no. 21 (2022): 1532–1540. 35857624 10.1097/BRS.0000000000004417 PMC9554757

[5] IBGE , “Pesquisa Nacional de Saúde,” 2015, https://www.pns.icict.fiocruz.br/.

[6] A. Singh , K. Lindsay Tetreault , S. Kalsi‐Ryan , A. Nouri , and M. G. Fehlings , “Global Prevalence and Incidence of Traumatic Spinal Cord Injury,” Clinical Epidemiology 6 (2014): 309–331. 25278785 10.2147/CLEP.S68889 PMC4179833

[7] R. A. Soares , F. B. Z. de Figueiredo , P. A. Leal Neto , et al., “Incidência de traumas raquimedulares causados por acidentes de trânsitos no nordeste de 2020 a 2022: Uma análise transversal,” Research, Society and Development 12, no. 3 (2023): e19412340623.

[8] NSCISC , “Traumatic Spinal Cord Injury Facts and Figures at a Glance,” 2024.

[9] M. Khorasanizadeh , M. Yousefifard , M. Eskian , et al., “Neurological Recovery Following Traumatic Spinal Cord Injury: A Systematic Review and Meta‐Analysis,” Journal of Neurosurgery. Spine 30 (2019): 683–699. 30771786 10.3171/2018.10.SPINE18802

[10] M. J. T. Sfair , “Effects of the Voluntary Exercise on the Recovery of Spinal Cord Hemissection: Changes in the Perineuronal Net and Histon Acetilation” (2014).

[11] A. McDonough and V. Martínez‐Cerdeño , “Endogenous Proliferation After Spinal Cord Injury in Animal Models,” Stem Cells International 2012 (2012): 387513. 23316243 10.1155/2012/387513 PMC3539424

[12] A. Alizadeh , S. M. Dyck , and S. Karimi‐Abdolrezaee , “Traumatic Spinal Cord Injury: An Overview of Pathophysiology, Models and Acute Injury Mechanisms,” Frontiers in Neurology 10 (2019): 282. 30967837 10.3389/fneur.2019.00282 PMC6439316

[13] M. F. Neves , J. L. R. Fonseca , P. C. S. Carvalho , et al., “Analysis of Movements in Spinal Cord Hemisection Treatment With Amniotic Membrane—Preclinical Study,” Asian Journal of Physical and Chemical Sciences 10 (2022): 28–37.

[14] C. Zhao , W. Song , J. S. Rao , et al., “Combination of Kinematic Analyses and Diffusion Tensor Tractrography to Evaluate the Residual Motor Functions in Spinal Cord‐Hemisected Monkeys,” Journal of Medical Primatology 46, no. 5 (2017): 239–247. 28543057 10.1111/jmp.12276

[15] R. J. Dumont , D. O. Okonkwo , S. Verma , et al., “Acute Spinal Cord Injury, Part I: Pathophysiologic Mechanisms,” Clinical Neuropharmacology 24 (2001): 254–264. 11586110 10.1097/00002826-200109000-00002

[16] T. B. Jones , E. E. Mcdaniel , and P. G. Popovich , “Inflammatory‐Mediated Injury and Repair in the Traumatically Injured Spinal Cord,” Current Pharmaceutical Design 11 (2005): 1223–1236. 15853679 10.2174/1381612053507468

[17] Y. Zou , “An Update on Spinal Cord Injury Research,” Neuroscience Bulletin 29, no. 4 (2013): 399–401. 23893427 10.1007/s12264-013-1366-3 PMC5561948

[18] C. S. Ahuja , S. Nori , L. Tetreault , et al., “Traumatic Spinal Cord Injury—Repair and Regeneration,” Clinical Neurosurgery 80, no. 3 (2017): S22–S90. 10.1093/neuros/nyw080 28350947

[19] M. Oudega , “Inflammatory Response After Spinal Cord Injury,” Experimental Neurology 250 (2013): 151–155. 24063891 10.1016/j.expneurol.2013.09.013

[20] I. Eli , D. P. Lerner , and Z. Ghogawala , “Acute Traumatic Spinal Cord Injury,” Neurologic Clinics 39, no. 2 (2021): 471–488. 33896529 10.1016/j.ncl.2021.02.004

[21] F. I. Khan and Z. Ahmed , “Experimental Treatments for Spinal Cord Injury: A Systematic Review and Meta‐Analysis,” Cells 11, no. 21 (2022): 3409. 36359804 10.3390/cells11213409 PMC9653737

[22] Z. Ara , S. Walliullah , D. Rastogi , S. Pandey , S. Pant , and R. M. Tripathi , “Effect of Various Treatment Modalities After Spinal Cord Injury,” Acta Scientific Orthopaedics 5 (2022): 56–73.

[23] C. Yao , X. Tang , Y. Cao , X. Wang , and B. Yu , “A Brief Summary of Current Therapeutic Strategies for Spinal Cord Injury,” Engineering 13 (2022): 46–52.

[24] M. R. Hamblin , “Shining Light on the Head: Photobiomodulation for Brain Disorders,” BBA Clinical 6 (2016): 113–124. 27752476 10.1016/j.bbacli.2016.09.002 PMC5066074

[25] H. Bendella , S. Rink , A. Wöhler , et al., “Anatomic Conditions for Bypass Surgery Between Rostral (T7–T9) and Caudal (L2, L4, S1) Ventral Roots to Treat Paralysis After Spinal Cord Injury,” Annals of Anatomy 222 (2018): 139–145. 30599238 10.1016/j.aanat.2018.12.008

[26] D. Cass , Steroids in Acute Spinal Cord Injury Canadian, (2017).

[27] C. M. Dumont , M. A. Carlson , M. K. Munsell , et al., “Aligned Hydrogel Tubes Guide Regeneration Following Spinal Cord Injury,” Acta Biomaterialia 86 (2019): 312–322. 30610918 10.1016/j.actbio.2018.12.052 PMC6369008

[28] V. C. Nogueira , N. P. M. de Freitas Coelho , T. L. de Barros , S. M. M. de Sousa Silva , M. Martins , and E. A. L. Arisawa , “Biomodulation Effects of LED and Therapeutic Ultrasound Combined With Semipermeable Dressing in the Repair Process of Cutaneous Lesions in Rats,” Acta Cirúrgica Brasileira 29, no. 9 (2014): 588–595. 25252205 10.1590/s0102-8650201400150006

[29] A. A. Paula , R. A. Nicolau , M. de Oliveira Lima , M. A. C. Salgado , and J. C. Cogo , “Low‐Intensity Laser Therapy Effect on the Recovery of Traumatic Spinal Cord Injury,” Lasers in Medical Science 29, no. 6 (2014): 1849–1859. 24858233 10.1007/s10103-014-1586-4

[30] M. Scandola , L. Dodoni , G. Lazzeri , et al., “Neurocognitive Benefits of Physiotherapy for Spinal Cord Injury,” Journal of Neurotrauma 36 (2018): 2028–2035. 10.1089/neu.2018.6123 30526335

[31] J. J. Anders , R. Lanzafame , and P. Arany , “Low‐Level Light/Laser Therapy Versus Photobiomodulation Therapy,” Photomedicine and Laser Surgery 33, no. 4 (2015): 183–184. 25844681 10.1089/pho.2015.9848 PMC4390214

[32] R. L. Marcos , M. M. Evaristo , P. de Almeida‐Mattos , et al., “Photobiomodulation Controls the Expression of Lipoxin Receptors, Promoting the Resolution of the Inflammatory Process in an Experimental Tendinitis Model,” Journal of Orthopaedic Research 43, no. 5 (2025): 1035–1044. 40045730 10.1002/jor.26063

[33] M. Neves , B. Souza , C. Souza , et al., “Comparative Analysis of Movements After Experimental Spinal Cord Injury Treated With Amniotic Membrane: Pilot Study,” Neurological Disorders and Therapeutics 2, no. 2 (2018): 1–5.

[34] L. Ramos , E. C. P. Leal Junior , R. C. Pallotta , et al., “Infrared (810 nm) Low‐Level Laser Therapy in Experimental Model of Strain‐Induced Skeletal Muscle Injury in Rats: Effects on Functional Outcomes,” Photochemistry and Photobiology 88 (2012): 154–160. 22053933 10.1111/j.1751-1097.2011.01030.x

[35] L. Ramos , R. L. Marcos , R. Torres‐Silva , et al., “Characterization of Skeletal Muscle Strain Lesion Induced by Stretching in Rats: Effects of Laser Photobiomodulation,” Photomedicine and Laser Surgery 36 (2018): 460–467. 30096269 10.1089/pho.2018.4473

[36] S. A. Dos Santos , M. A. Dos Santos Vieira , M. C. B. Simões , A. J. Serra , E. C. Leal‐Junior , and P. T. C. de Carvalho , “Photobiomodulation Therapy Associated With Treadmill Training in the Oxidative Stress in a Collagen‐Induced Arthritis Model,” Lasers in Medical Science 32, no. 5 (2017): 1071–1079. 28429194 10.1007/s10103-017-2209-7

[37] M. V. da Silva Leal , M. O. Lima , D. R. Costa , et al., “Evaluation of the Effects of Photobiomodulation (808 nm) on Pain and Quality of Life of Diabetic Neuropathy Patients,” Research, Society and Development 11, no. 2 (2022): e26211225552.

[38] D. R. Pessoa , D. R. Costa , B. de Moraes Prianti , et al., “Association of Facial Massage, Dry Needling, and Laser Therapy in Temporomandibular Disorder: Case Report,” Codas 30, no. 6 (2018): e20170265. 30517267 10.1590/2317-1782/20182017265

[39] M. A. Naeser and M. R. Hamblin , “Traumatic Brain Injury: A Major Medical Problem That Could be Treated,” Photomedicine and Laser Surgery 33, no. 9 (2015): 443–446. 26280257 10.1089/pho.2015.3986 PMC4560854

[40] L. Yang , C. Wu , E. Parker , et al., “Non‐Invasive Photobiomodulation Treatment in an Alzheimer Disease‐Like Transgenic Rat Model,” Theranostics 12, no. 5 (2022): 2205–2231. 35265207 10.7150/thno.70756 PMC8899582

[41] S. Xu , W. Zhu , M. Shao , et al., “Ecto‐5′‐Nucleotidase (CD73) Attenuates Inflammation After Spinal Cord Injury by Promoting Macrophages/Microglia M2 Polarization in Mice,” Journal of Neuroinflammation 15, no. 1 (2018): 155. 29788960 10.1186/s12974-018-1183-8 PMC5964922

[42] J. Zhang , J. Sun , Q. Zheng , et al., “Low‐Level Laser Therapy 810‐nm Up‐Regulates Macrophage Secretion of Neurotrophic Factors via PKA‐CREB and Promotes Neuronal Axon Regeneration In Vitro,” Journal of Cellular and Molecular Medicine 24, no. 1 (2020): 476–487. 31667932 10.1111/jcmm.14756 PMC6933332

[43] A. L. Drozdov , T. I. Karu , V. M. Chudnovskii , V. I. Yusupov , and V. N. Bagratashvili , “Influence of Low‐Intensity Red Diode and Laser Radiation on the Locomotor Activity of Sea Urchin Sperm,” Doklady. Biochemistry and Biophysics 457, no. 1 (2014): 146–148. 25172337 10.1134/S1607672914040085

[44] X. Li , X.‐K. Wang , Z.‐J. Zhu , et al., “Photobiomodulation Provides Neuroprotection Through Regulating Mitochondrial Fission Imbalance in the Subacute Phase of Spinal Cord Injury,” Neural Regeneration Research 9, no. 9 (2023): 2005–2010. 10.4103/1673-5374.366491 PMC10233780 36926726

[45] J. Yang , L. Wang , and M. X. Wu , “830 nm Photobiomodulation Therapy Promotes Engraftment of Human Umbilical Cord Blood‐Derived Hematopoietic Stem Cells,” Scientific Reports 10, no. 1 (2020): 19671. 33184429 10.1038/s41598-020-76760-5 PMC7661704

[46] R. C. Mosca , A. A. Ong , O. Albasha , K. Bass , and P. Arany , “Photobiomodulation Therapy for Wound Care: A Potent, Noninvasive, Photoceutical Approach,” Advances in Skin & Wound Care 32, no. 4 (2019): 157–167. 30889017 10.1097/01.ASW.0000553600.97572.d2

[47] J. Fedorova and J. Pavel , “An Accurate Method for Histological Determination of Neural Tissue Loss/Sparing After Compression‐Induced Spinal Cord Injury With Optimal Reproducibility,” Journal of Neurotrauma 36, no. 18 (2019): 2665–2675. 30648463 10.1089/neu.2018.6140

[48] L. Karatanasov , L. Borges , D. Campos , et al., “Kinematic and Sensory‐Motor Analysis of the Effects of Treatments With Photobiomodulation in Rats With Experimentally Induced Spinal Injury,” Lasers in Medical Science 40, no. 1 (2025): 134. 40064714 10.1007/s10103-025-04399-7

[49] A. Falavigna , F. Cechetti , G. Finger , L. G. Ruschel , G. Marcon , and P. G. da Silva , “Experimental Model of Spinal Cord Injury (SCI) in Rats: Management Guidelines,” Coluna/Columna 12 (2013): 70–72.

[50] D. Correia , L. Lima , L. Sant'anna , M. Lima , and E. Arisawa , “Histological Analysis of Spinal Cord Injury Treated With Amniotic Membrane,” Medicina 58 (2025): e222620.

[51] S. B. Damy , R. S. Camargo , R. Chammas , and L. F. P. de Figueiredo , “Aspectos fundamentais da experimentação animal—aplicações em cirurgia experimental,” Revista Da Associacao Medica Brasileira 56, no. 1 (2010): 103–111. 20339795 10.1590/s0104-42302010000100024

[52] F. Ramezani , M. Razmgir , K. Tanha , et al., “Photobiomodulation for Spinal Cord Injury: A Systematic Review and Meta‐Analysis,” Physiology & Behavior 224 (2020): 112977. 32504695 10.1016/j.physbeh.2020.112977

[53] J. C. Fleming , M. D. Norenberg , D. A. Ramsay , et al., “The Cellular Inflammatory Response in Human Spinal Cords After Injury,” Brain 129, no. 12 (2006): 3249–3269. 17071951 10.1093/brain/awl296

[54] T. M. O'Shea , J. E. Burda , and M. V. Sofroniew , “Cell Biology of Spinal Cord Injury and Repair,” Journal of Clinical Investigation 127, no. 9 (2017): 3259–3270. 28737515 10.1172/JCI90608 PMC5669582

[55] M. K. Giacci , L. Wheeler , S. Lovett , et al., “Differential Effects of 670 and 830 nm Red Near Infrared Irradiation Therapy: A Comparative Study of Optic Nerve Injury, Retinal Degeneration, Traumatic Brain and Spinal Cord Injury,” PLoS One 9, no. 8 (2014): e104565. 25105800 10.1371/journal.pone.0104565 PMC4126771

[56] J. W. Song , K. Li , Z. W. Liang , et al., “Low‐Level Laser Facilitates Alternatively Activated Macrophage/Microglia Polarization and Promotes Functional Recovery After Crush Spinal Cord Injury in Rats,” Scientific Reports 7, no. 1 (2017): 620. 28377600 10.1038/s41598-017-00553-6 PMC5428709

[57] J. Chedly , S. Soares , A. Montembault , et al., “Physical Chitosan Microhydrogels as Scaffolds for Spinal Cord Injury Restoration and Axon Regeneration,” Biomaterials 138 (2017): 91–107. 28554011 10.1016/j.biomaterials.2017.05.024

[58] D. J. Hellenbrand , C. M. Quinn , Z. J. Piper , C. N. Morehouse , J. A. Fixel , and A. S. Hanna , “Inflammation After Spinal Cord Injury: A Review of the Critical Timeline of Signaling Cues and Cellular Infiltration,” Journal of Neuroinflammation 18, no. 1 (2021): 284. 34876174 10.1186/s12974-021-02337-2 PMC8653609

[59] T. Karu , “Photobiology of Low‐Power Laser Effects,” Health Physics 56, no. 5 (1989): 691–704. 2651364 10.1097/00004032-198905000-00015

[60] M. S. Pedram , M. M. Dehghan , M. Shojaee , et al., “Therapeutic Effects of Simultaneous Photobiomodulation Therapy (PBMT) and Meloxicam Administration on Experimental Acute Spinal Cord Injury: Rat Animal Model,” Journal of Photochemistry and Photobiology. B 189 (2018): 49–54. 10.1016/j.jphotobiol.2018.09.022 30312920

[61] A. Shuaib and A. K. Bourisly , “Photobiomodulation Optimization for Spinal Cord Injury Rat Phantom Model,” Translational Neuroscience 9, no. 1 (2018): 67–71. 29967691 10.1515/tnsci-2018-0012 PMC6024694

[62] S. Veronez , L. Assis , P. del Campo , et al., “Effects of Different Fluences of Low‐Level Laser Therapy in an Experimental Model of Spinal Cord Injury in Rats,” Lasers in Medical Science 32, no. 2 (2017): 343–349. 27909916 10.1007/s10103-016-2120-7

[63] M. R. Hamblin , “Mechanisms and Applications of the Anti‐Inflammatory Effects of Photobiomodulation,” AIMS Biophysics 4, no. 3 (2017): 337–361. 28748217 10.3934/biophy.2017.3.337 PMC5523874

[64] S.‐Y. Chang and M. Y. Lee , “Photobiomodulation of Neurogenesis Through the Enhancement of Stem Cell and Neural Progenitor Differentiation in the Central and Peripheral Nervous Systems,” International Journal of Molecular Sciences 24, no. 20 (2023): 15427. 37895108 10.3390/ijms242015427 PMC10607539

[65] M. C. Nicodemo , E. A. L. S. Arisawa , L. B. Sant'anna , and R. Lopes‐Martins , “Photobiomodulation and Amniotic Membrane for Treat Tendon Injury in Rats,” Anais da Academia Brasileira de Ciências 96, no. S1 (2024): e20231139. 39140521 10.1590/0001-3765202420231139

[66] J. A. F. Santos , R. A. Nicolau , L. B. Sant'Anna , et al., “Diabetic Foot Wounds Treated With Human Amniotic Membrane and Low‐Level Laser Therapy: A Pilot Clinical Study,” Wound Management & Prevention 67, no. 8 (2021): 16–23. 34370677

